# Bioorthogonal chemical imaging of metabolic activities in live mammalian hippocampal tissues with stimulated Raman scattering

**DOI:** 10.1038/srep39660

**Published:** 2016-12-21

**Authors:** Fanghao Hu, Michael R. Lamprecht, Lu Wei, Barclay Morrison, Wei Min

**Affiliations:** 1 Department of Chemistry, Columbia University, New York, New York, USA; 2 Department of Biomedical Engineering, Columbia University, New York, New York, USA; 3 Kavli Institute for Brain Science, Columbia University, New York, New York, USA

## Abstract

Brain is an immensely complex system displaying dynamic and heterogeneous metabolic activities. Visualizing cellular metabolism of nucleic acids, proteins, and lipids in brain with chemical specificity has been a long-standing challenge. Recent development in metabolic labeling of small biomolecules allows the study of these metabolisms at the global level. However, these techniques generally require nonphysiological sample preparation for either destructive mass spectrometry imaging or secondary labeling with relatively bulky fluorescent labels. In this study, we have demonstrated bioorthogonal chemical imaging of DNA, RNA, protein and lipid metabolism in live rat brain hippocampal tissues by coupling stimulated Raman scattering microscopy with integrated deuterium and alkyne labeling. Heterogeneous metabolic incorporations for different molecular species and neurogenesis with newly-incorporated DNA were observed in the dentate gyrus of hippocampus at the single cell level. We further applied this platform to study metabolic responses to traumatic brain injury in hippocampal slice cultures, and observed marked upregulation of protein and lipid metabolism particularly in the hilus region of the hippocampus within days of mechanical injury. Thus, our method paves the way for the study of complex metabolic profiles in live brain tissue under both physiological and pathological conditions with single-cell resolution and minimal perturbation.

On the physiology level, brain activities comprise both relatively fast chemical (calcium and neurotransmitter) or electrical signals and relatively slow metabolic turnover of small metabolites into DNA, RNA, proteins and lipids. While the former signaling process has been extensively probed by electrophysiology and fluorescent techniques^1^,^2^,^3^, visualizing the overall downstream genetic replication, transcription, translation and lipid metabolism in brain with cellular resolution has been a long-standing challenge. Biomacromolecules including nucleic acids, proteins and lipids are made of repeated small-molecule building blocks either derived from glucose glycolysis intermediates or directly from nutrients like amino acids, choline, fatty acids and nucleosides. Thus, labeling through small building blocks allows the visualization of cellular metabolism at a global level^4^,^5^,^6^,^7^,^8^,^9^,^10^,^11^,^12^,^13^,^14^. However, these metabolically labeled species either rely on destructive detection based on mass spectrometry, or lack other intrinsic imaging contrast, thereby requiring cell fixation and secondary labeling with relatively bulky fluorescent labels. Novel imaging methods that accomplish the direct metabolic imaging in living systems would enable researchers the unprecedented ability to map out distributions and to follow dynamics of these important metabolic activities in the brain tissue.

As an emerging multiphoton optical imaging technique, stimulated Raman scattering (SRS) microscopy is sensitive and specific in detecting chemical bonds, offering diffraction-limited subcellular resolution, linear concentration dependence with background-free chemical contrast for quantitative measurement and intrinsic 3D optical sectioning capability^15^,^16^,^17^,^18^,^19^,^20^,^21^,^22^. The use of picosecond excitation pulses and near-infrared wavelength also reduce photon scattering inside tissue samples and potential phototoxicity^23^,^24^,^25^. Recently, two small bioorthogonal labels (alkyne and carbon-deuterium bonds) have been developed for SRS microscopy. Bioorthogonal chemical imaging of small biomolecules with these two labels has been separately demonstrated in live cells, including amino acids, nucleic acids, glycan, choline, fatty acids, glucose and cholesterol^26^,^27^,^28^,^29^,^30^,^31^,^32^,^33^,^34^,^35^. To harness both the strong Raman signal of alkyne^36^,^37^,^38^ and the high incorporation efficiency of deuterium^26^,^31^,^39^ for SRS imaging, we rationally integrate alkyne and deuterium labeling according to the biochemical property of individual species. For the first time we demonstrate this integrated platform in visualizing multiple metabolisms in the brain tissue including a traumatic brain injury model (Fig. 1a).

The mammalian hippocampus is an active region of brain metabolism involved in learning, memory formation, and has strong implications in many neurological diseases. Organotypic hippocampal slice culture provides a complex tissue model that mimics the structure and activity *in vivo* with multiple cells types (neurons and glia) coexisting in a three-dimensional architecture^40^. It is known to have well-preserved structural and functional characteristics, with clear identification of neuronal regions of dentate gyrus (DG), cornu ammonis 3 (CA3) and CA1 interconnected by the Mossy fibers and Schaffer collaterals^41^. In addition, as compared to acute slice culture that can only last for days in our previous report^30^, organotypic hippocampal tissue slices can be maintained for several weeks, allowing for long-term study, and are suitable for studying tissue metabolism with a relatively slow turnover. By coupling SRS microscopy with integrated deuterium and alkyne labels, we can visualize active metabolisms of DNA, RNA, protein and lipids in both the dentate gyrus and CA regions of rat hippocampus. Characteristic patterns of metabolic incorporation are observed for different metabolite species. By targeting DNA synthesis, newly-generated neurons are highlighted at the single cell level, and symmetric neural precursor cell division is observed in the subgranular zone of the dentate gyrus, where active neurogenesis occurs.

Traumatic brain injury (TBI) is one of the leading causes of adult disability and death worldwide. It commonly occurs during sports (concussions) and is also prevalent among youth, being the primary cause of death in childhood^42^,^43^,^44^. It is caused by rapid deformation of the brain during trauma including impact, penetration or deceleration forces, such as in automobile accidents or falls, which can lead to a cascade of pathological events at the molecular level and ultimately neurodegeneration. The hippocampus is commonly found damaged in TBI from post-mortem and *in vivo* studies^45^,^46^. Many studies have reported the changes in the long-term potentiation and calcium flux in hippocampal neurons after mechanical injury^47^,^48^,^49^,^50^. However, how these acute responses influence the downstream metabolism of the hippocampus and how the hippocampal tissue responds to the resulting injury metabolically remains poorly understood.

Therefore, visualizing hippocampus metabolism before and after mechanical trauma will identify the key region involved in tissue damage and degeneration and provide potential target for selective intervention. In this study, a previously developed *in vitro* TBI model is used to simulate the mechanical deformation experienced by the brain tissue during *in vivo* trauma^51^,^52^. We found that, compared to uninjured tissues, rat hippocampal tissues undergoing mechanical stretch injury exhibit markedly increased protein and lipid synthesis within days of injury, indicating fast and activated anabolic metabolism for cell proliferation and repair. Therefore, our method provides an important demonstration of probing metabolic responses in a traumatic brain injury model by bioorthogonal chemical imaging.

## Results

### Visualization of protein and lipid metabolism in live rat hippocampal tissues

**Figure 1:**
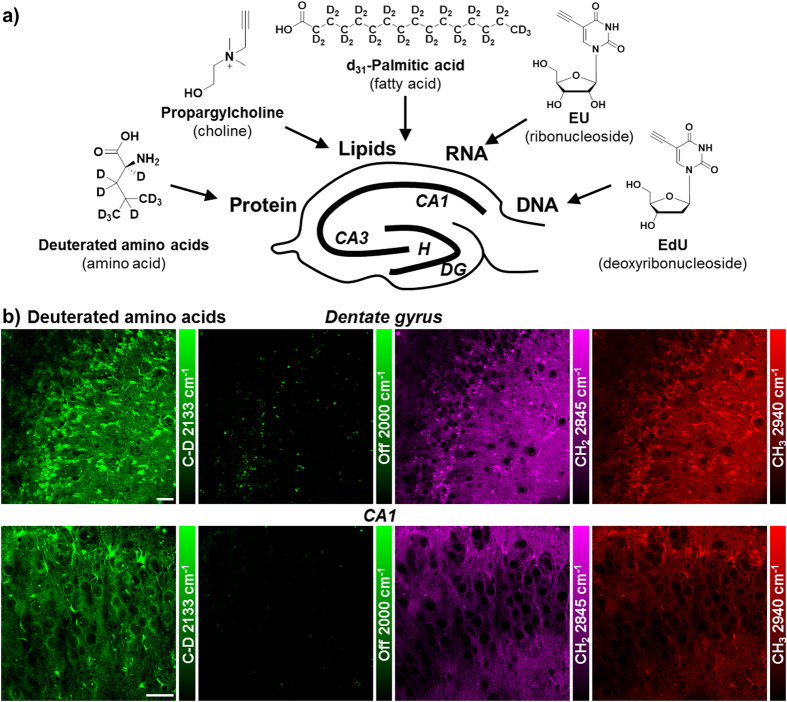
Metabolic labeling of hippocampus with integrated alkyne and deuterium labeled small metabolites for SRS microscopy. (**a**) Chemical structures of alkyne or deuterium labeled molecules used in the study and illustration of mammalian hippocampal structure indicated with DG, CA3, CA1 and H (hilus) regions. Deuterated amino acids are represented by d_10_-Leucine. (**b**) Protein metabolism in live rat hippocampal slices with deuterated amino acids. Active amino acid incorporation and protein synthesis are imaged in both dentate gyrus and CA1 regions of hippocampus. The C-D images at 2133 cm^−1^ show the newly synthesized protein from deuterated amino acids. The 2000 cm^−1^ images are the off-resonance background channels of the same area. The CH_2_ 2845 cm^−1^ and CH_3_ 2940 cm^−1^ channels show the intrinsic distribution of total lipids and proteins in the same region. Rat hippocampal slices are cultured in media supplemented with deuterated amino acids for 3 days before SRS imaging. Scale bar: 40 μm.

Due to the high incorporation efficiency of deuterated amino acids than the alkyne-labeled counterparts, they are used to visualize protein metabolism in live rat hippocampal slices with SRS microscopy. Figure 1b shows active amino acid incorporation and new protein synthesis throughout the hippocampus including both dentate gyrus and CA regions. The images at 1900 or 2000 cm^−1^ show the off-resonance signal as the background reference. And the CH_2_ vibrations at 2845 cm^−1^ mainly from total lipids and CH_3_ vibrations at 2940 cm^−1^ mainly from total proteins are imaged label-free to outline the cell morphology in tissue.

**Figure 2:**
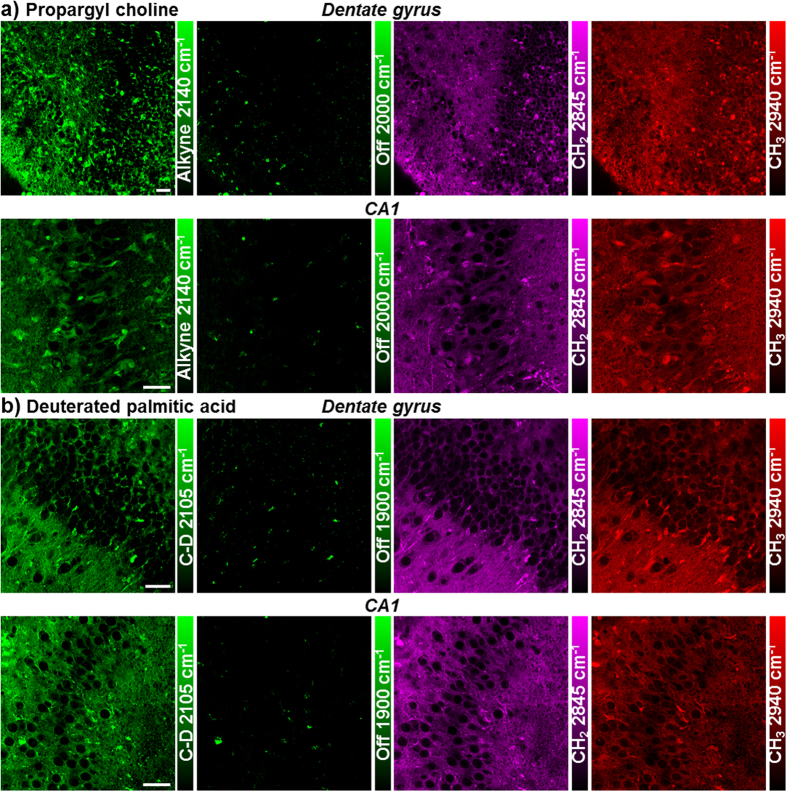
Lipid metabolism in live rat hippocampal slices with propargyl choline and fully deuterated palmitic acid (d_31_-PA). Active membrane synthesis and lipid metabolism from both choline and fatty acid are imaged in both dentate gyrus and CA1 regions of hippocampus. The alkyne images at 2140 cm^−1^ show the newly incorporated phospholipids with propargyl choline and the C-D images at 2105 cm^−1^ show the newly synthesized lipids from d_31_-PA. The 1900 and 2000 cm^−1^ channels are the off-resonance images of the same area. The CH_2_ 2845 cm^−1^ and CH_3_ 2940 cm^−1^ channels are label-free images of total lipids and proteins in the same region. (**a**) Rat hippocampal slices are incubated with 2 mM propargyl choline for 2–3 days before SRS imaging. (**b**) Rat hippocampal slices are incubated with 200 μM d_31_-PA for 5 days before SRS imaging. Scale bar: 40 μm.

Choline is the hydrophilic headgroup of phosphatidylcholine, which is a highly abundant phospholipid in cell membrane. With the strong signal of the alkyne label, propargyl choline^10^ is applied to visualize phospholipid turnover and cellular membrane synthesis (Fig. 2a). Fatty acid is a common precursor for many lipids with the hydrophobic acyl chain, and fully deuterated palmitic acid (d_31_-PA) is used to visualize lipid synthesis and metabolism in live rat hippocampal tissues (Fig. 2b). New membrane synthesis and lipid incorporation from both choline and fatty acid are imaged with strong intensity in both dentate gyrus and CA regions of hippocampus. Overall, with deuterated amino acids, propargyl choline and d_31_-PA as metabolic building blocks, organotypic hippocampal tissues from neonatal rats are shown to have globally active metabolism of proteins and lipids, which is successfully imaged by SRS microscopy coupled with small bioorthogonal vibrational labels.

### Metabolic patterns of amino acids, choline and fatty acid

**Figure 3:**
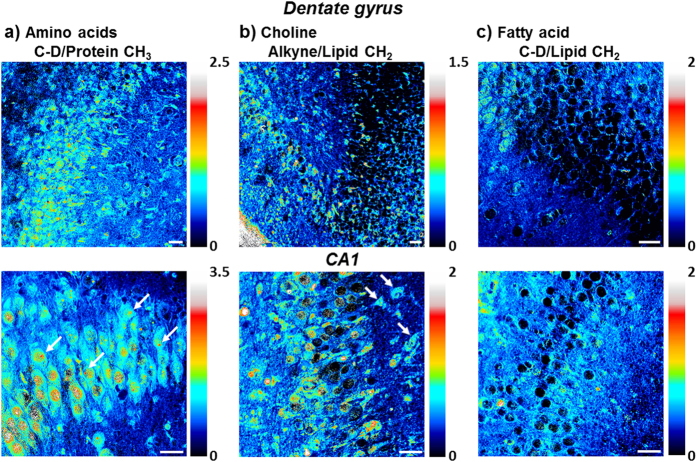
Metabolic incorporation patterns of different metabolite species from quantitative ratiometric images. (**a**) Ratiometric images of new protein synthesis (C-D 2133 cm^−1^) over old proteins (CH_3_ 2940 cm^−1^) in both dentate gyrus and CA1 regions of hippocampus. Protein synthesis is highly active in the neuronal cell body particularly the nucleoli (arrows). (**b**) Ratiometric images of new choline phospholipid synthesis (alkyne 2140 cm^−1^) over old lipids (CH_2_ 2845 cm^−1^) in both dentate gyrus and CA1 regions of hippocampus. Choline is enriched in soma structures of selected cells (arrows). (**c**) Ratiometric images of fatty acid incorporation and new lipid synthesis (C-D 2105 cm^−1^) over old lipids (CH_2_ 2845 cm^−1^) in both dentate gyrus and CA1 regions of hippocampus. Palmitic acid is evenly incorporated in the hippocampal tissues. Scale bar: 40 μm.

A more quantitative examination in the incorporation pattern of each metabolite by dividing over the label-free images of lipid or protein distribution reveals characteristic metabolic patterns of amino acids, choline and fatty acid (Fig. 3). In protein metabolism, new protein synthesis is the most active in the neuronal cell body, with strong signal in both the granule cell layer of DG and pyramidal cells in CA1 region. Protein turnover is especially fast in the nucleoli, which lights up inside the nucleus with predominant newly-synthesized protein from ribosomal biogenesis (Fig. 3a).

For phospholipid metabolism, the incorporation of choline headgroup into phospholipids is favored in selected cells and sparsely labeled in both the dentate gyrus and CA1 region of the hippocampal tissues. At the single-cell level, choline is enriched into the soma structure outside nucleus, highlighting the individual cell body out of the surrounding lipid-rich region (Fig. 3b). In general, protein and phospholipid turnover are shown to be faster in the cell-body-rich grey matter region than in the extended processes, with higher ratios of newly synthesized protein and choline phospholipids (Fig. 3a,b).

In fatty acid metabolism probed with deuterated palmitic acid, newly synthesized lipids from palmitic acid incorporation are more evenly distributed in the tissue, with much less variation in the ratios across the tissue in both the cell body and the processes except the nucleus (Fig. 3c), compared to the protein and phospholipid distribution. This suggests a more uniform incorporation and metabolism of palmitic acid as a common intermediate of lipid species and biomass in cells.

### Nucleic acid metabolism and cell division dynamics in live rat hippocampal tissues

**Figure 4:**
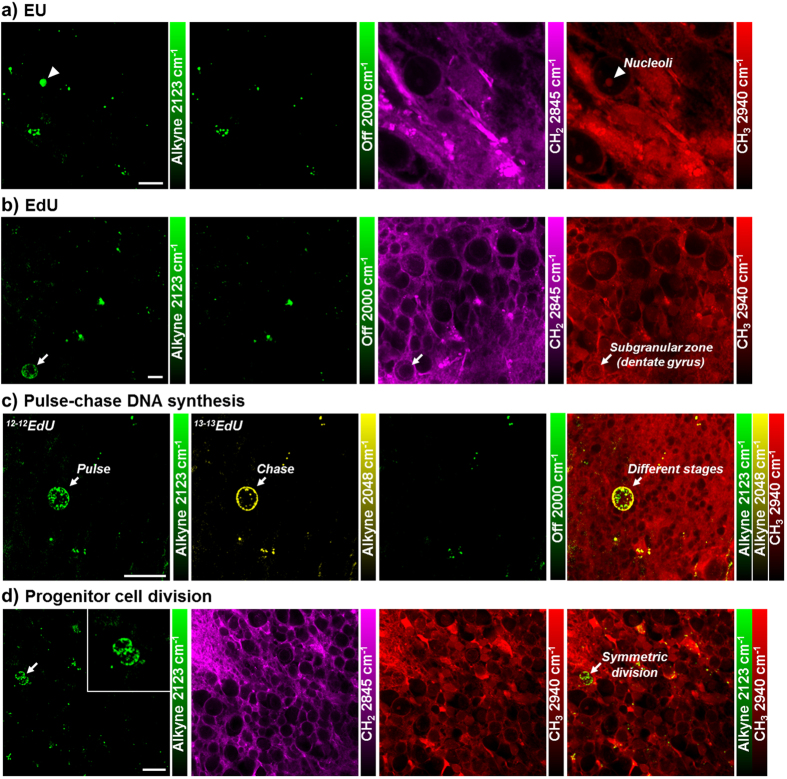
Nucleic acid metabolism and cell division dynamics in live rat hippocampal slices with ethynyl uridine (EU) and ethynyl deoxyuridine (EdU). (**a**) Newly synthesized RNA is visualized in the nucleoli of CA1 pyramidal neurons. Rat hippocampal slices are incubated with 2 mM EU (alkyne 2123 cm^−1^) for 7 hours before SRS imaging. (**b**) Newly synthesized DNA is imaged inside the nucleus of neuron at the subgranular zone of dentate gyrus, indicating neurogenesis. Rat hippocampal slices are incubated with 300 μM EdU (alkyne 2123 cm^−1^) for 3 days before SRS imaging. (**a,b**) Scale bar: 10 μm. (**c**) Different DNA incorporation patterns are observed with two-color pulse-chase labeling of EdU, likely from two cell division cycles. Rat hippocampal slices are pulsed with 400 μM ^12-12^EdU (alkyne 2123 cm^−1^) for 12 hours then chased with 400 μM ^13-13^EdU (alkyne 2048 cm^−1^) for 14 hours before SRS imaging. (**d**) Symmetric neural progenitor cell division is captured in the dentate gyrus of hippocampal tissue. Inset: zoom-in area showing the distribution of newly synthesized DNA. Rat hippocampal slices are incubated with 200 μM EdU for 3 days before SRS imaging. (**c,d**) Scale bar: 20 μm.

Genetic material is the origin of cellular metabolism. Nucleic acid metabolism is also visualized in live rat hippocampal slices using alkyne labeled nucleosides. Newly synthesized RNA is imaged using ethynyl uridine (EU)^8^ in the CA1 pyramidal neuron, which accumulates in the nucleoli that is known to have a high concentration and turnover of ribosome RNA (Fig. 4a). Moreover, with ethynyl deoxyuridine (EdU)^9^, newly synthesized DNA for new cell division is visualized inside the nucleus at the single cell level in the subgranular zone of granule cell layer (GCL) (Fig. 4b), demonstrating active neurogenesis in postnatal rat hippocampus development.

Previously, our group has developed ^13^C isotope edited alkyne label for multicolor SRS imaging of different species in cell culture^53^. Here we apply both ^12^C and ^13^C labeled EdU isotopologues to follow DNA synthesis dynamics in precursor cell division by two-color pulse-chase labeling (Fig. 4c). We first pulse the rat hippocampal tissue with ^12^C labeled EdU for 12 hours and then chase with ^13^C labeled EdU with 14 hours. Distinct patterns of metabolically labeled DNA are observed at comparable intensity in the same cell, resulting from different time points of synthesis and incorporation. The early incorporated DNA in green is more evenly distributed in the nucleus while the lately incorporated DNA in yellow is concentrated in a ring structure at the outer edge of the nucleus, suggesting a mid-S phase in the current cell cycle.

Symmetric cell division with mirror-image like distribution of newly synthesized DNA is also captured in the dentate gyrus of live rat hippocampal tissue (Fig. 4d and inset), suggesting neural progenitor cell division. Further, by optically sectioning the tissue at different depths with SRS, multiple cells with newly incorporated DNA are observed in the same region of the dentate gyrus (Fig. S1), indicating frequent neurogenesis. Many newly born cells migrate from the hilus into the granule cell layer during postnatal development^54^,^55^. Based on the relative distance of the newborn cells from the interface between the granule cell layer and the hilus of the dentate gyrus, the relative age of these new granule cells within the 3-day labeling window could be estimated. The hilar region is circled in red and characterized by the high content of axons. The neurons marked by EdU deep in the granule cell layer are likely to occur earlier than the ones closer to the hilar region.

### Metabolic responses in live rat hippocampal tissues after traumatic injury

**Figure 5:**
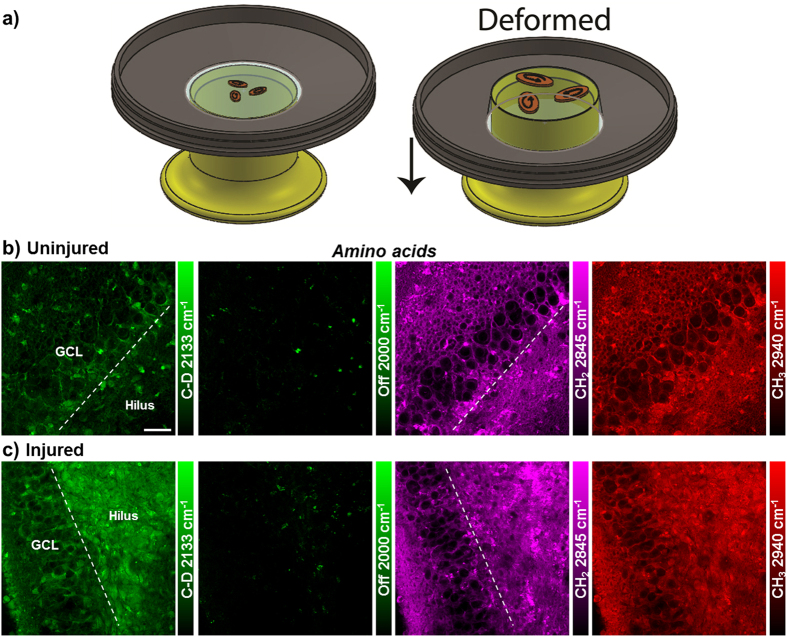
Metabolic response in live rat hippocampal slices after traumatic injury. **(a)** Schematic diagram depicting three slice cultures on a silicone membrane held in a stainless steel well for inducing mechanical deformation. The well is displaced over a circular indenter to generate an equi-biaxial and spatially homogenous strain field on the membrane and the adhered tissues. **(b,c)** Increased amino acid incorporation and protein synthesis are observed in the hilus of dentate gyrus. Rat hippocampal slices without (**b**) or with (**c**) mechanical stretch are cultured in media supplemented with deuterated amino acids for 1 day before SRS imaging. Scale bar: 40 μm.

After characterizing metabolic activities of rat hippocampus under physiological conditions, we next studied the metabolic responses in live rat hippocampal slices after traumatic injury. Live rat hippocampal slices are cultured on a deformable membrane, to which an equi-biaxial and spatially homogenous strain field is applied with active feedback control to stretch the tissue accurately and reproducibly (Fig. 5a)^51^,^52^. Because brain tissue is an incompressible material, this two-dimensional mechanical stretch of organotypic hippocampal slice induces tissue compression in the orthogonal direction, mimicking the three-dimension state of strain and tissue deformation caused by deceleration forces^52^,^56^. In this *in vitro* TBI model, we can simulate the tissue injury occurred during *in vivo* brain trauma such as automobile accidents or falls and monitor the resulting metabolic changes by SRS microscopy.

**Figure 6:**
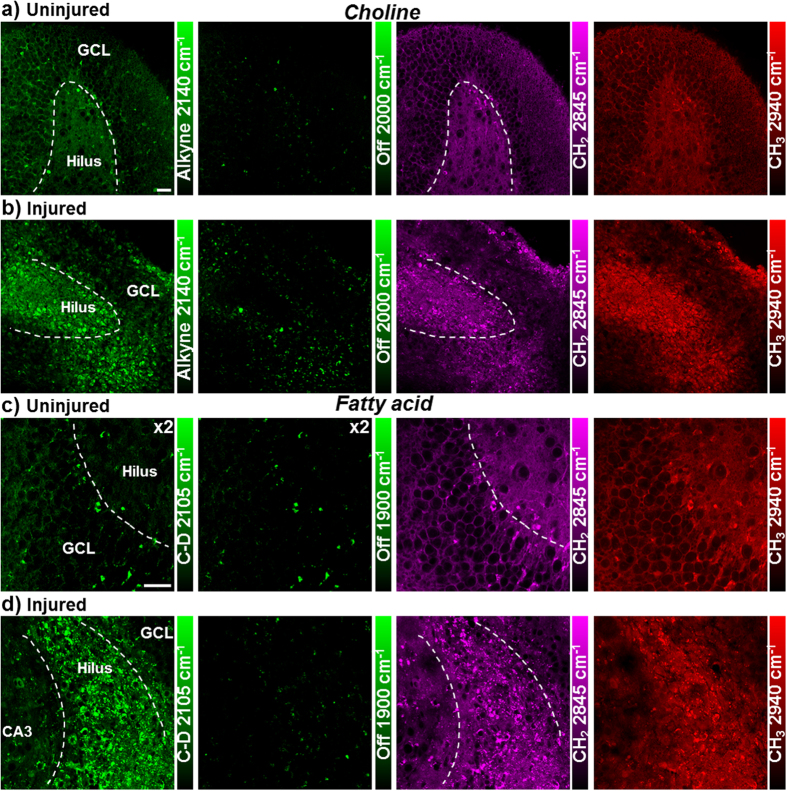
Upregulated lipid metabolism in live rat hippocampal slices after stretch injury. Increased choline and fatty acid metabolism for membrane synthesis are observed in the hilus of dentate gyrus. (**a,b**) Rat hippocampal slices without (**a**) or with (**b**) mechanical stretch are incubated with 2 mM propargyl choline for 2 days before SRS imaging. (**c,d**) Rat hippocampal slices without (**c**) or with (**d**) mechanical stretch are incubated with 100 μM d_31_-PA for 1 day before SRS imaging. Scale bar: 40 μm.

Strong metabolic responses are observed in the stretch injured rat hippocampal tissues within days of injury. By imaging protein metabolism with deuterated amino acid, an increase in new protein synthesis is visualized in live rat hippocampal slices one day after the stretch injury, compared to uninjured hippocampal tissues (Fig. 5b,c). The increase in protein synthesis is especially prominent (3-fold) in the hilar region of dentate gyrus (Supplementary Fig. S2). Similarly, lipid synthesis and metabolism from both propargyl choline and deuterated palmitic acid incorporation are also found to be enhanced in the injured hippocampal tissues, particularly in the hilar region of the dentate gyrus (Fig. 6). Especially for the fatty acid, which is a key intermediate for cell lipid synthesis and cell proliferation, an over 10 times increase in the signal is observed in the hilus of the injured tissues after one day (Fig. 6c,d and Supplementary Fig. S2). Overall, this shows wide-range metabolic responses in both protein and lipid metabolism of hippocampal tissues undergoing mechanical injury. Amino acids, choline and fatty acid provide the important metabolic building blocks in cell biomass synthesis and accumulation for fast cell repair and proliferation in short time.

## Discussion

We have presented complex metabolic imaging in live mammalian hippocampal tissue by SRS microscopy coupled with integrated alkyne and deuterium bioorthogonal labels. By labeling with amino acids, choline, fatty acid and nucleosides, protein, lipids, RNA and DNA metabolism have been visualized in rat organotypic hippocampal slices, where active incorporation and metabolism occur throughout the dentate gyrus and CA regions. As a major step further from the previous cell culture studies, this work demonstrates the utility of bioorthogonal chemical imaging with SRS microscopy to map out complex activities and dynamics of brain metabolism in organotypic tissue culture.

Characteristic spatial patterns of metabolic incorporation are observed for amino acids, choline and fatty acid, indicative of key locations of synthesis and degradation of each species. Protein synthesis and turnover is highly active in the neuronal cell body especially in the nucleoli (Fig. 3a), whereas lipid synthesis is most abundant outside the nucleus with choline incorporation being concentrated in the soma structure surrounding the nucleus (Fig. 3b) and fatty acid being uniformly incorporated throughout the hippocampal tissues (Fig. 3c). These observations are consistent with the subcellular active sites of fast protein turnover in nucleoli^57^, phospholipid synthesis on endoplasmic reticulum^58^ and fatty acid utilization as a general metabolic intermediate in cellular biomass synthesis.

As the center of memory in brain, genetic material turnover and neurogenesis are especially important in hippocampus metabolism. Ribosome RNA transcription is visualized in the nucleoli of CA1 pyramidal neurons (Fig. 4a). Newly generated neurons are identified at the single-cell level with metabolically labeled DNA in the subgranular zone of rat hippocampal tissues (Fig. 4b). Using two-color pulse-chase labeling to follow the cell division cycle with isotope-edited EdU, different patterns of DNA synthesis and incorporation are shown, suggesting two cell cycles (Fig. 4c). The time interval between consecutive division cycles of the same cell can be estimated to be less than 26 hours, which is consistent with the cell cycle length (25 hours) of cells in the dentate gyrus of rat hippocampus^59^. In addition, symmetric progenitor cell division is captured in the subgranular zone of dentate gyrus (Fig. 4d), and multiple neurons with newly synthesized DNA are visualized in the same region of granule cell layer at different depths with 3D optical sectioning of SRS (Fig. S1). The relative origin of time point of these cells could be inferred from their distances with reference to the granule cell layer and hilus of the dentate gyrus. This highlights the multiple approaches applicable for following DNA metabolism, particularly key to the neurogenesis in the hippocampus.

Visualizing the metabolic changes in hippocampus under pathological condition is also achieved in an *in vitro* TBI model, which mimics *in vivo* brain trauma during car accidents or falls. Strongly increased protein and lipid synthesis are observed in stretch injured tissues within 1–2 days of trauma, especially in the hilus of the hippocampus (Figs 5 and 6 and Supplementary Fig. S2). This could be due to both increase in cell density and local elevation of cellular metabolic activities. The hilar region of the dentate gyrus is rich in axon projections originated from granule cell layer and mossy cells^60^. Thus, it could be most sensitive to mechanical force and responds strongly to the resulting axonal injury and mossy fiber disruption by activating a cascade of anabolic metabolism for cell regeneration and neuron repair.

In conclusion, we have integrated the bioorthogonal chemical labels and demonstrated their application in visualizing complex metabolism in live rat hippocampal tissues with SRS microscopy. Our technique is capable to monitor specific metabolic activities of DNA, RNA, protein and lipids in complex cellular settings and sensitively capture the metabolic changes occurred during tissue damage in a traumatic brain injury model. The same platform can be potentially applied to other brain disease or injury models based on organotypic brain tissue culture. It is expected that our technique will be a powerful tool to visualize global downstream metabolism after initial signaling events and to identify key regions of interest for therapeutic development in treating concussion-related head injury, especially if combined with small molecule screening^61^.

## Methods

### Stimulated Raman scattering microscopy

All laser beams are produced by a custom-modified laser system (picoEMERALD, Applied Physics & Electronics, Inc.) A fundamental 1064 nm Stokes laser (6 ps pulse width) is generated at 80 MHz repetition rate, and its intensity is modulated sinusoidally by an electro-optic-modulator at 8 MHz with >90% modulation depth. A mode-locked pump beam (5–6 ps pulse width) is produced by a build-in optical parametric oscillator to have a tunable range of 720–990 nm. Both laser beams are coupled into an inverted laser-scanning multiphoton microscope (FV1200MPE, Olympus) with optimized near-IR throughput. The spatial and temporal overlapping of pump and Stokes beam are achieved using two dichroic mirrors and a delay stage inside the laser system based on the heavy water SRS signal. A 25× water objective (XLPlan N, 1.05 N.A. MP, Olympus) with high near-IR transmission is used to image all samples. The beam sizes of pump and Stokes laser are adjusted to match the back-aperture of the objective. After the sample in the forward-transmitted direction, a high N.A. condenser lens (oil immersion, 1.4 N.A., Olympus) collects both beams in Kohler illumination with high efficiency. Beam motion from laser-scanning is descanned with a telescope and a high O.D. bandpass filter (890/220 CARS, Chroma Technology) is used to block the Stokes beam completely and only passes the pump beam. A large-area (10 mm × 10 mm) silicon photodiode (FDS1010, Thorlabs) is reverse-biased with a 64 DC voltage to maximize saturation threshold and response bandwidth and is used to collect the entire pump beam. The output photocurrent is electronically filtered to remove both the 80 MHz component of laser pulsing and low frequency fluctuations from scanning motion using a 8 MHz electronic bandpass filter (KR 2724, KR electronics), and is terminated with 50 Ω before entering a radio frequency lock-in amplifier (SR844, Stanford Research Systems). The corresponding voltage signal is demodulated at the reference frequency to extract the stimulated Raman loss signal from the pump beam with near short-noise-limited sensitivity. SRS images are generated by inputting the in-phase signal at the X channel of the lock-in amplifier to the analog interface box (FV10-ANALOG) of the microscope at each pixel and scanning across the whole field of view. ~120 mW pump beam and ~150 mW modulated Stokes beam, measured after the 25× water objective, are used to image the sample at all frequencies. The demodulation time constant is 30 μs and the imaging pixel dwell time is 100 μs with ~26 s/frame (512 × 512 pixels) for all images.

### Organotypic hippocampal slice culture

Organotypic hippocampal slice culture (OHSC) is performed as described in previous literature^41^,^52^ and the protocol is approved by IACUC at Columbia University. All experiments are performed in accordance with the relevant guidelines and regulations. In brief, Sprague Dawley rat pups are decapitated at postnatal day 8–10 (P8–P10), and the hippocampus is quickly isolated and placed in ice-cold Gey’s balanced salt solution (Sigma). A McIlwain tissue chopper is used to cut the hippocampus into 400 μm thick sections which are immediately plated on Millicell cell culture inserts (Millipore) or silicone membranes in Neurobasal (Invitrogen) medium supplemented with B27 (1X, Invitrogen), GlutaMAX (1 mM, Invitrogen), and D-glucose (4.5 mg/ml, Sigma) at 37 °C and 5% CO_2_. After 2 days *in vitro* (DIV), the medium is changed to medium containing serum comprised of 50% MEM (Sigma), 25% heat-inactivated horse serum (Sigma), 25% Hank’s balanced salt solution (Sigma), GlutaMAX (1 mM, Invitrogen), and D-glucose (4.5 mg/ml, Sigma). Medium is changed every 2–3 days.

### *In vitro* model of traumatic brain injury

After 10 DIV, OHSCs cultured on silicone membranes are subjected to a moderate mechanical injury, known to induce changes in gene expression consistent with an *in vivo* injury^62^. The injury is induced by stretching the underlying silicone substrate to a predetermined strain (20%) at a predetermined strain rate under motion-control to produce the desired tissue injury. Our well-established model produces a highly accurate and reproducible injury to OHSCs^40^,^52^,^56^. High speed video (MotionPro, Redlake, Pasadena, CA) is used to verify tissue deformation by image analysis at 1000 frames per second. Lagrangian strain of the tissue is determined by calculating the deformation gradient tensor by locating fiduciary markers on the tissue slice before and at maximal stretch using custom MATLAB (MathWorks, Natick, MA) scripts^61^.

### Metabolic incorporation of alkyne and deuterium labeled species

After 7–12 DIV in regular medium or immediately after injury, OHSC medium is changed to fresh culture medium supplemented with deuterated amino acids, propargyl choline, d_31_-PA (Sigma), EU (Invitrogen) or EdU (Invitrogen) at specified concentrations for the given time. A full recipe of deuterated amino acids medium is given in the supporting information adapted from previous literature^30^. At the end of the incubation, the tissue slices are washed with phosphate buffered saline (PBS, Sigma) for 3 times and transferred into a chamber filled with PBS solution ready for SRS imaging.

### Imaging processing

All images were collected with FluoView scanning software, assigned color and analyzed by ImageJ. Ratiometric images are calculated with ImageJ under identical parameters.

## Additional Information

**How to cite this article**: Hu, F. *et al*. Bioorthogonal chemical imaging of metabolic activities in live mammalian hippocampal tissues with stimulated Raman scattering. *Sci. Rep.* **6**, 39660; doi: 10.1038/srep39660 (2016).

**Publisher's note:** Springer Nature remains neutral with regard to jurisdictional claims in published maps and institutional affiliations.

## Supplementary Material

Supplementary Information

## References

[b1] Zhang J., Campbell R. E., Ting A. Y. & Tsien R. Y. Creating new fluorescent probes for cell biology. Nat. Rev. Mol. Cell Biol. 3, 906–918 (2002). 12461557 10.1038/nrm976

[b2] Yuste R. (ed) Imaging: A Laboratory Manual. (Cold Spring Harbor Press, 2010).

[b3] Berenyi A. . Large-scale, high-density (up to 512 channels) recording of local circuits in behaving animals. J. Neurophysiol. 111, 1132–1149 (2014). 24353300 10.1152/jn.00785.2013 PMC3949233

[b4] Prescher J. A. & Bertozzi C. R. Chemistry in living systems. Nat. Chem. Biol. 1, 13–21 (2005). 16407987 10.1038/nchembio0605-13

[b5] Grammel M. & Hang H. C. Chemical reporters for biological discovery. Nat. Chem. Biol. 9, 475–484 (2013). 23868317 10.1038/nchembio.1296 PMC3866016

[b6] Beatty K. E. . Fluorescence visualization of newly synthesized proteins in mammalian cells. Angew. Chem. Int. Ed. Engl. 45, 7364–7367 (2006). 17036290 10.1002/anie.200602114

[b7] Baskin J. M. . Copper-free click chemistry for dynamic *in vivo* imaging. Proc. Natl. Acad. Sci. USA 104, 16793–16797 (2007). 17942682 10.1073/pnas.0707090104 PMC2040404

[b8] Jao C. Y. & Salic A. Exploring RNA transcription and turnover *in vivo* by using click chemistry. Proc. Natl. Acad. Sci. USA 105, 15779–15784 (2008). 18840688 10.1073/pnas.0808480105 PMC2572917

[b9] Salic A. & Mitchison T. J. A chemical method for fast and sensitive detection of DNA synthesis *in vivo*. Proc. Natl. Acad. Sci. USA 105, 2415–2420 (2008). 18272492 10.1073/pnas.0712168105 PMC2268151

[b10] Jao C. Y., Roth M., Welti R. & Salic A. Metabolic labeling and direct imaging of choline phospholipids *in vivo*. Proc. Natl. Acad. Sci. USA 106, 15332–15337 (2009). 19706413 10.1073/pnas.0907864106 PMC2741251

[b11] Hang H. C., Wilson J. P. & Charron G. Bioorthogonal chemical reporters for analyzing protein lipidation and lipid trafficking. Acc. Chem. Res. 44, 699–708 (2011). 21675729 10.1021/ar200063v PMC4231477

[b12] Lechene C. . High-resolution quantitative imaging of mammalian and bacterial cells using stable isotope mass spectrometry. J. Biol. 5, 20 (2006). 17010211 10.1186/jbiol42 PMC1781526

[b13] Zhang D. S. . Multi-isotope imaging mass spectrometry reveals slow protein turnover in hair-cell stereocilia. Nature 481, 520–524 (2012). 22246323 10.1038/nature10745 PMC3267870

[b14] Yuet K. P. & Tirrell D. A. Chemical tools for temporally and spatially resolved mass spectrometry-based proteomics. Ann. Biomed. Eng. 42, 299–311 (2014). 23943069 10.1007/s10439-013-0878-3 PMC3925203

[b15] Freudiger C. W. . Label-free biomedical imaging with high sensitivity by stimulated Raman scattering microscopy. Science 322, 1857–1861 (2008). 19095943 10.1126/science.1165758 PMC3576036

[b16] Min W., Freudiger C. W., Lu S. & Xie X. S. Coherent nonlinear optical imaging: beyond fluorescence microscopy. Annu. Rev. Phys. Chem. 62, 507–530 (2011). 21453061 10.1146/annurev.physchem.012809.103512 PMC3427791

[b17] Suhalim J. L., Boik J. C., Tromberg B. J. & Potma E. O. The need for speed. J. Biophotonics 5, 387–395 (2012). 22344721 10.1002/jbio.201200002 PMC3383092

[b18] Zhang D., Wang P., Slipchenko M. N. & Cheng J.-X. Fast vibrational imaging of single cells and tissues by stimulated Raman scattering microscopy. Acc. Chem. Res. 47, 2282–2290 (2014). 24871269 10.1021/ar400331q PMC4139189

[b19] Cheng J.-X. & Xie X. S. Vibrational spectroscopic imaging of living systems: An emerging platform for biology and medicine. Science 350, aaa8870 (2015). 26612955 10.1126/science.aaa8870

[b20] Wei L. . Live-Cell Bioorthogonal Chemical Imaging: Stimulated Raman Scattering Microscopy of Vibrational Probes. Acc. Chem. Res. 49, 1494–1502 (2016). 27486796 10.1021/acs.accounts.6b00210 PMC5704954

[b21] Camp C. H. & Cicerone M. T. Chemically sensitive bioimaging with coherent Raman scattering. Nat. Photon. 9, 295–305 (2015).

[b22] Krafft C., Schie I. W., Meyer T., Schmitt M. & Popp J. Developments in spontaneous and coherent Raman scattering microscopic imaging for biomedical applications. Chem. Soc. Rev. 45, 1819–1849 (2016). 26497570 10.1039/c5cs00564g

[b23] Pezacki J. P. . Chemical contrast for imaging living systems: molecular vibrations drive CARS microscopy. Nat. Chem. Biol. 7, 137–145 (2011). 21321552 10.1038/nchembio.525 PMC7098185

[b24] Ozeki Y. . High-speed molecular spectral imaging of tissue with stimulated Raman scattering. Nat. Photon. 6, 844–850 (2012).

[b25] Ji M. . Detection of human brain tumor infiltration with quantitative stimulated Raman scattering microscopy. Sci. Transl. Med. 7, 309ra163 (2015). 10.1126/scitranslmed.aab0195 PMC4900155 26468325

[b26] Wei L., Yu Y., Shen Y., Wang M. C. & Min W. Vibrational imaging of newly synthesized proteins in live cells by stimulated Raman scattering microscopy. Proc. Natl. Acad. Sci. USA 110, 11226–11231 (2013). 23798434 10.1073/pnas.1303768110 PMC3710790

[b27] Wei L. . Live-cell imaging of alkyne-tagged small biomolecules by stimulated Raman scattering. Nature Methods 11, 410–412 (2014). 24584195 10.1038/nmeth.2878 PMC4040164

[b28] Hong S. . Live-cell stimulated Raman scattering imaging of alkyne-tagged biomolecules. Angew. Chem. Int. Ed. Engl. 53, 5827–5831 (2014). 24753329 10.1002/anie.201400328

[b29] Hu F., Wei L., Zheng C., Shen Y. & Min W. Live-cell vibrational imaging of choline metabolites by stimulated Raman scattering coupled with isotope-based metabolic labeling. Analyst 139, 2312–2317 (2014). 24555181 10.1039/c3an02281a PMC4069604

[b30] Wei L. . Imaging complex protein metabolism in live organisms by stimulated Raman scattering microscopy with isotope labeling. ACS Chem. Biol. 10, 901–908 (2015). 25560305 10.1021/cb500787b PMC4610303

[b31] Zhang D., Slipchenko M. N. & Cheng J.-X. Highly Sensitive Vibrational Imaging by Femtosecond Pulse Stimulated Raman Loss. J. Phys. Chem. Lett. 2, 1248–1253 (2011). 21731798 10.1021/jz200516n PMC3124560

[b32] Li J. & Cheng J.-X. Direct visualization of de novo lipogenesis in single living cells. Sci. Rep. 4, 6807 (2014). 25351207 10.1038/srep06807 PMC4212242

[b33] Hu F. . Vibrational Imaging of Glucose Uptake Activity in Live Cells and Tissues by Stimulated Raman Scattering. Angew. Chem. Int. Ed. Engl. 54, 9821–9825 (2015). 26207979 10.1002/anie.201502543 PMC4644272

[b34] Lee H. J. . Assessing cholesterol storage in live cells and C. elegans by stimulated Raman scattering imaging of phenyl-Diyne cholesterol. Sci. Rep. 5, 7930 (2015). 25608867 10.1038/srep07930 PMC4302291

[b35] Alfonso-Garcia A., Pfisterer S. G., Riezman H., Ikonen E. & Potma E. O. D38-cholesterol as a Raman active probe for imaging intracellular cholesterol storage. J. Biomed. Opt. 21, 61003 (2016). 26719944 10.1117/1.JBO.21.6.061003 PMC4681884

[b36] Weeks T., Wachsmann-Hogiu S. & Huser T. Raman microscopy based on doubly-resonant four-wave mixing (DR-FWM). Opt. Express 17, 17044–17051 (2009). 19770922 10.1364/OE.17.017044

[b37] Yamakoshi H. . Imaging of EdU, an alkyne-tagged cell proliferation probe, by Raman microscopy. J. Am. Chem. Soc. 133, 6102–6105 (2011). 21443184 10.1021/ja108404p

[b38] Yamakoshi H. . Alkyne-tag Raman imaging for visualization of mobile small molecules in live cells. J. Am. Chem. Soc. 134, 20681–20689 (2012). 23198907 10.1021/ja308529n

[b39] Fu D. . *In vivo* metabolic fingerprinting of neutral lipids with hyperspectral stimulated Raman scattering microscopy. J. Am. Chem. Soc. 136, 8820–8828 (2014). 24869754 10.1021/ja504199s PMC4073829

[b40] Sundstrom L., Morrison B., Bradley M. & Pringle A. Organotypic cultures as tools for functional screening in the CNS. Drug Discov. Today 10, 993–1000 (2005). 16023058 10.1016/S1359-6446(05)03502-6

[b41] Stoppini L., Buchs P. A. & Muller D. A simple method for organotypic cultures of nervous tissue. J. Neurosci. Methods 37, 173–182 (1991). 1715499 10.1016/0165-0270(91)90128-m

[b42] Laker S. R. Epidemiology of concussion and mild traumatic brain injury. PM R 3, S354–358 (2011). 22035677 10.1016/j.pmrj.2011.07.017

[b43] Meaney D. F., Morrison B. & Dale Bass C. The mechanics of traumatic brain injury: a review of what we know and what we need to know for reducing its societal burden. J. Biomech. Eng. 136, 021008 (2014). 24384610 10.1115/1.4026364 PMC4023660

[b44] Thurman D. J. The Epidemiology of Traumatic Brain Injury in Children and Youths: A Review of Research Since 1990. J. Child Neurol. 31, 20–27 (2016). 25123531 10.1177/0883073814544363

[b45] Tate D. F. & Bigler E. D. Fornix and hippocampal atrophy in traumatic brain injury. Learn. Mem. 7, 442–446 (2000). 11112803 10.1101/lm.33000

[b46] Lifshitz J. . Structural and functional damage sustained by mitochondria after traumatic brain injury in the rat: evidence for differentially sensitive populations in the cortex and hippocampus. J. Cereb. Blood Flow Metab. 23, 219–231 (2003). 12571453 10.1097/01.WCB.0000040581.43808.03

[b47] Miyazaki S. . Enduring suppression of hippocampal long-term potentiation following traumatic brain injury in rat. Brain Res. 585, 335–339 (1992). 1511317 10.1016/0006-8993(92)91232-4

[b48] D’Ambrosio R., Maris D. O., Grady M. S., Winn H. R. & Janigro D. Selective loss of hippocampal long-term potentiation, but not depression, following fluid percussion injury. Brain Res. 786, 64–79 (1998). 9554957 10.1016/s0006-8993(97)01412-1

[b49] Lusardi T. A., Wolf J. A., Putt M. E., Smith D. H. & Meaney D. F. Effect of acute calcium influx after mechanical stretch injury *in vitro* on the viability of hippocampal neurons. J. Neurotrauma 21, 61–72 (2004). 14987466 10.1089/089771504772695959

[b50] Vogel E. W. 3rd . Isolated Primary Blast Inhibits Long-Term Potentiation in Organotypic Hippocampal Slice Cultures. J. Neurotrauma 33, 652–661 (2016). 26414012 10.1089/neu.2015.4045 PMC5583564

[b51] Cater H. L., Sundstrom L. E. & Morrison B. Temporal development of hippocampal cell death is dependent on tissue strain but not strain rate. J Biomech. 39, 2810–2818 (2006). 16289515 10.1016/j.jbiomech.2005.09.023

[b52] Morrison B. 3rd, Cater H. L., Benham C. D. & Sundstrom L. E. An *in vitro* model of traumatic brain injury utilising two-dimensional stretch of organotypic hippocampal slice cultures. J. Neurosci. Methods 150, 192–201 (2006). 16098599 10.1016/j.jneumeth.2005.06.014

[b53] Chen Z. . Multicolor live-cell chemical imaging by isotopically edited alkyne vibrational palette. J. Am. Chem. Soc. 136, 8027–8033 (2014). 24849912 10.1021/ja502706q PMC4063185

[b54] Schlessinger A. R., Cowan W. M. & Gottlieb D. I. An autoradiographic study of the time of origin and the pattern of granule cell migration in the dentate gyrus of the rat. J. Comp. Neurol. 159, 149–175 (1975). 1112911 10.1002/cne.901590202

[b55] Cameron H. A., Woolley C. S., McEwen B. S. & Gould E. Differentiation of newly born neurons and glia in the dentate gyrus of the adult rat. Neuroscience 56, 337–344 (1993). 8247264 10.1016/0306-4522(93)90335-d

[b56] Cater H. L. . Stretch-induced injury in organotypic hippocampal slice cultures reproduces *in vivo* post-traumatic neurodegeneration: role of glutamate receptors and voltage-dependent calcium channels. J. Neurochem. 101, 434–447 (2007). 17250683 10.1111/j.1471-4159.2006.04379.x

[b57] Andersen J. S. . Nucleolar proteome dynamics. Nature 433, 77–83 (2005). 15635413 10.1038/nature03207

[b58] Farooqui A. A., Horrocks L. A. & Farooqui T. Glycerophospholipids in brain: their metabolism, incorporation into membranes, functions, and involvement in neurological disorders. Chem. Phys. Lipids 106, 1–29 (2000). 10878232 10.1016/s0009-3084(00)00128-6

[b59] Cameron H. A. & McKay R. D. Adult neurogenesis produces a large pool of new granule cells in the dentate gyrus. J. Comp. Neurol. 435, 406–417 (2001). 11406822 10.1002/cne.1040

[b60] Scharfman H. E. & Myers C. E. Hilar mossy cells of the dentate gyrus: a historical perspective. Front. Neural Circuits 6, 106 (2012). 23420672 10.3389/fncir.2012.00106 PMC3572871

[b61] Lamprecht M. R. & Morrison B. 3rd A Combination Therapy of 17beta-Estradiol and Memantine Is More Neuroprotective Than Monotherapies in an Organotypic Brain Slice Culture Model of Traumatic Brain Injury. J. Neurotrauma 32, 1361–1368 (2015). 25752651 10.1089/neu.2015.3912

[b62] Lamprecht M. R. . Strong Correlation of Genome-Wide Expression after Traumatic Brain Injury *In Vitro* and *In Vivo* Implicates a Role for SORLA. J. Neurotrauma doi: 10.1089/neu.2015.4306 (2016). PMC5198072 26919808

