# Supplementary material for: Bioorthogonal chemical imaging of metabolic activities in live mammalian hippocampal tissues with stimulated Raman scattering
Source: Sci Rep. 2016 Dec 21;6:39660. doi: 10.1038/srep39660 (PMC5175176; doi:10.1038/srep39660)
Supplement: Supplementary Information [file srep39660-s1.pdf]

Bioorthogonal chemical imaging of metabolic activities in live mammalian hippocampal tissues with stimulated Raman scattering

Fanghao Hu[1], Michael R. Lamprecht[2], Lu Wei[1], Barclay Morrison III[2]* and Wei Min[1,3]*

1. Department of Chemistry, Columbia University, New York, New York, USA

2. Department of Biomedical Engineering, Columbia University, New York, New York, USA

3. Kavli Institute for Brain Science, Columbia University, New York, New York, USA

Correspondence to bm2119@columbia.edu or wm2256@columbia.edu

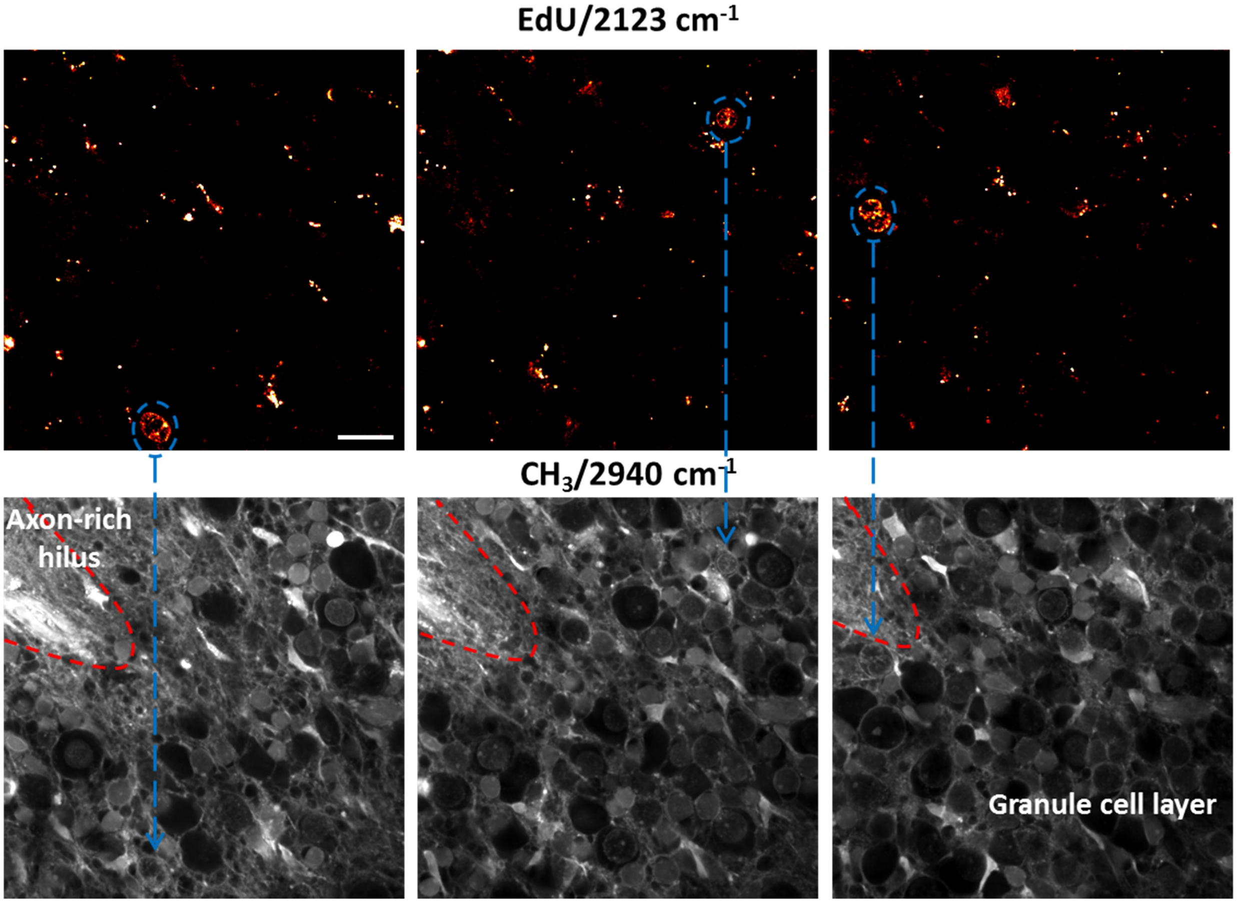

Supplementary Figure S1. Frequent neurogenesis in the dentate gyrus of live rat hippocampal slice. Multiple newly generated granule cells are observed in the same region of dentate gyrus in z sectioning images of every 5 μm. Rat hippocampal slices are incubated with 200 μM EdU for 3 days before SRS imaging. Scale bar: 20 μm.

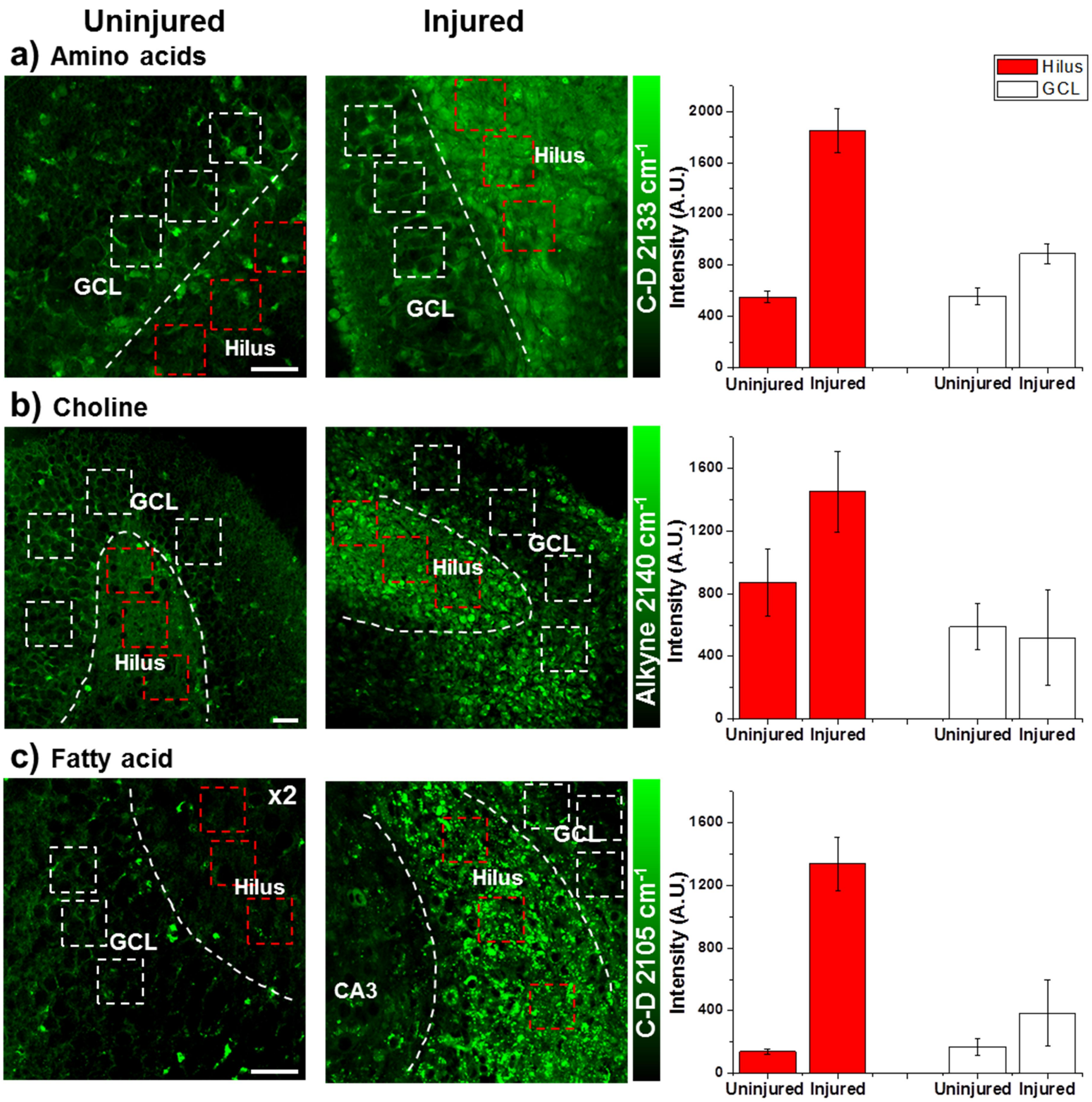

Supplementary Figure S2. Increased protein and lipid metabolism in live rat hippocampal tissues after mechanical injury. (a) A 3-fold increase in protein synthesis is observed in the hilar region of the injured tissue. (b) A 1.7 times increase in choline incorporation is observed in the hilar region of the injured tissue. (c) An over 10-fold increase in fatty acid incorporation is observed in the hilus of the injured tissue. Increase protein and lipid metabolism are also observed in the granule cell layer (GCL) of dentate gyrus, but the extent of metabolic response is weaker. Data are shown as mean ± standard deviation. Scale bar: 40 μm.

Deuterated amino acids medium for metabolic labeling of organotypic hippocampal tissue
The culture medium is made of deuterated Neurobasal-A medium (shown below as described in previous literature) with 1X B27 serum-free supplement (Invitrogen), 1 mM GlutaMAX (Invitrogen) and 4.5 mg/ml D-glucose (Sigma).

Amino acids	Concentration (mM)	Catalog number and product company
Glycine-d_5	0.4	DLM-280, Cambridge isotope
L-Alanine-d_4	0.022	DLM-250, Cambridge isotope
L-Arginine·HCl-d_7	0.398	DLM-541, Cambridge isotope
L-Asparagine-d_8	0.006	672947 ALDRICH (Isotech)
L-Cysteine·2HCl (regular)	0.26	C6727, SIGMA
L-Histidine·HCl·H_2O (regular)	0.2	H5659, SIGMA
L-Isoleucine-d_{10}	0.802	DLM-141, Cambridge isotope
L-Leucine-d_{10}	0.802	DLM-567, Cambridge isotope
L-Lysine·HCl-d_8	0.798	616214, ALDRICH (Isotech)
L-Methionine-d_3	0.201	DLM-431, Cambridge isotope
L-Phenylalanine-d_8	0.4	DLM-372, Cambridge isotope
L-Proline-d_7	0.067	DLM-487, Cambridge isotope
L-Serine-d_3	0.4	DLM-582, Cambridge isotope
L-Threonine (regular)	0.798	T8441, SIGMA
L-Tryptophan (regular)	0.078	T8941, SIGMA
L-Tyrosine-d_2	0.398	DLM-2317, Cambridge isotope
L-Valine-d_8	0.803	DLM-488, Cambridge isotope
The formulations of vitamins, inorganic salts and other components (without phenol red) are the same as in the regular Neurobasal-A medium (10888, Invitrogen).		
